# The Emerging Role of Electrophiles as a Key Regulator for Endoplasmic Reticulum (ER) Stress

**DOI:** 10.3390/ijms20071783

**Published:** 2019-04-10

**Authors:** Nobumasa Takasugi, Hideki Hiraoka, Kengo Nakahara, Shiori Akiyama, Kana Fujikawa, Ryosuke Nomura, Moeka Furuichi, Takashi Uehara

**Affiliations:** Department of Medicinal Pharmacology, Graduate School of Medicine, Dentistry, and Pharmaceutical Sciences, Okayama University, Okayama 700-8530, Japan; ntakasu@okayama-u.ac.jp (N.T.); p4dz03d5@s.okayama-u.ac.jp (H.H.); p4c57y1g@s.okayama-u.ac.jp (K.N.); prjy22tg@s.okayama-u.ac.jp (S.A.); ph426128@s.okayama-u.ac.jp (K.F.); p01107rk@s.okayama-u.ac.jp (R.N.); pqus4tlp@s.okayama-u.ac.jp (M.F.)

**Keywords:** reactive electrophiles, ER stress, UPR, nitric oxide, 4-hydroxynonenal, methylmercury

## Abstract

The unfolded protein response (UPR) is activated by the accumulation of misfolded proteins in the endoplasmic reticulum (ER), which is called ER stress. ER stress sensors PERK, IRE1, and ATF6 play a central role in the initiation and regulation of the UPR; they inhibit novel protein synthesis and upregulate ER chaperones, such as protein disulfide isomerase, to remove unfolded proteins. However, when recovery from ER stress is difficult, the UPR pathway is activated to eliminate unhealthy cells. This signaling transition is the key event of many human diseases. However, the precise mechanisms are largely unknown. Intriguingly, reactive electrophilic species (RES), which exist in the environment or are produced through cellular metabolism, have been identified as a key player of this transition. In this review, we focused on the function of representative RES: nitric oxide (NO) as a gaseous RES, 4-hydroxynonenal (HNE) as a lipid RES, and methylmercury (MeHg) as an environmental organic compound RES, to outline the relationship between ER stress and RES. Modulation by RES might be a target for the development of next-generation therapy for ER stress-associated diseases.

## 1. Endoplasmic Reticulum (ER) Stress and Electrophiles

The ER is a fundamental intracellular organelle that plays a key role in protein production, folding, and homeostasis. When protein folding is impaired, misfolded proteins accumulate in the ER. In the course of this accumulation, “ER stress” is induced, and the unfolded protein response (UPR) is activated. The UPR is an adaptive system designed to handle misfolded proteins in the ER [1]. The UPR promotes the folding or removal of unfolded proteins by translational repression, transcriptional activation of ER chaperones, and ER-associated degradation (ERAD) [2]. However, under excess or chronic ER stress, the UPR shifts signaling from the protective pathway to the apoptotic pathway, to eliminate unhealthy cells. Although disruption of these pathways is related to many human diseases, such as cancers, diabetes, and neurodegenerative diseases, when and how this shift in signaling takes place remain largely unclear.

Reactive electrophile species (RES) are molecules that have the ability to attract electrons to bond to nucleophiles—molecules with the property of supplying an electron pair [3]. RES, which exist in the environment or are generated by cellular metabolism, covalently bond to their targets, such as DNA and proteins, to alter their structures and physiological functions [4]. As described in the following sections, most RES modifications are reversible and occur in response to the surrounding environment, and RES can also regulate physiological conditions that are essential for maintaining homeostasis. However, when the balance of these mechanisms is disrupted, a pathological state is formed. Malignant neoplasms, diabetes mellitus, and neurodegenerative diseases, including Alzheimer’s disease (AD), Parkinson’s disease (PD), and amyotrophic lateral sclerosis (ALS), have been reported as candidate diseases involving RES. The relationship between these diseases and RES is reviewed elsewhere [5,6,7,8].

Intriguingly, it has been gradually identified that modulation of ER stress by RES is a critical pathogenic event for these diseases [9,10]. In this review, we discuss the modulation of ER stress by representative RES and provide an overview of their relationship and association with human diseases. We begin with the most analyzed gaseous RES, nitric oxide (NO), followed by lipid RES, 4-hydroxynonenal (HNE), and finally discuss one of the most critical environmental organic compounds RES, methylmercury (MeHg). 

## 2. NO and ER Stress

### 2.1. Physiological Properties and Functions of NO

NO is a gaseous molecule with a short half-life that can easily pass through the plasma membrane to regulate various cellular responses, such as apoptosis, proliferation, and neurotransmission [11,12,13,14,15,16,17]. Some of these events play essential roles in biological processes, such as the regulation of blood pressure and memory formation [18,19,20]. NO is intracorporeally synthesized from L-arginine by nitric oxide synthases (NOS), which consist of three isoforms: neuronal NOS (nNOS, NOS1), inducible NOS (iNOS, NOS2), and endothelial NOS (eNOS, NOS3) [21,22]. nNOS and eNOS are characterized by tissue-specific localization, whereas iNOS is broadly expressed in response to inflammation [22]. iNOS is upregulated by bacterial pathogens or immunostimulating cytokines and generates high NO concentrations. 

By reacting with oxygen and water, NO is metabolized into nitrite and nitrate. NO and these metabolites are called reactive nitrogen species and have free oxygen radicals, which cause high electrophilic reactivity [12,23,24,25]. NO attacks the cysteine thiol of various proteins to form *S*-nitrosothiol [26,27]. A target cysteine of *S*-nitrosylation has been suggested to be the thiolate anion, which is located at the acid–base motif [28,29]. This modification changes the enzyme activity, stability, or localization of target proteins [30]. Some NO functions have oppositional effects [19,31]. Appropriate amounts of NO contribute to neurotransmitter release or biophylaxis against viral or bacterial infection [32,33,34,35,36,37]. However, excessive amounts of NO cause abnormal modification of proteins, which are not targeted at steady-state concentrations [8,38,39,40,41,42].

**Figure 1:**
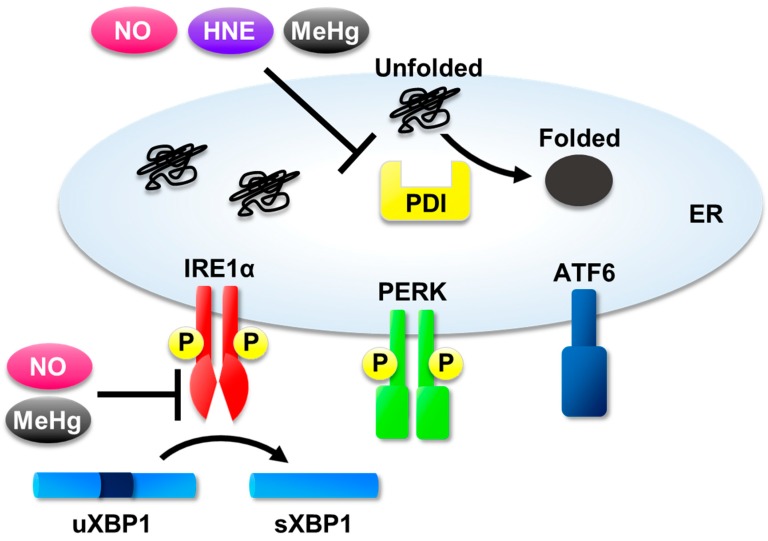
Targets of electrophiles in the unfolded protein response (UPR). NO, nitric oxide; HNE, 4-hydroxynonenal; MeHg, methylmercury; P, phosphate group; top arrow, protein folding process by PDI; lower arrow, RNA splicing process by IRE1α.

Interestingly, NO differentially modulates ER homeostasis through *S*-nitrosylation of targeted proteins related to the UPR, such as protein disulfide isomerase (PDI) and inositol-requiring kinase 1 alpha (IRE1α) (Figure 1). Here, we describe the association between NO-induced ER stress and various diseases.

### 2.2. PDI as a Target for NO

PDI is an ER chaperone that is mainly localized in the ER and plays a crucial role in protein folding through disulfide bond formation [43]. PDI is comprised of four domains, a, a’, b, and b’ domains, and the C-terminal region, which contains many acidic amino acids. The a and a’ domains are activation domains, which are separated by two domains, b and b’ [44]. The activation domains have an active site that consists of four amino acids (Cys–Gly–His–Cys). The oxidoreductase activity of PDI is derived from thiol groups of active site cysteines [45], and each active site (CGHC) is an oxidizing agent in the ER because of the high disulfide reduction potential and low pK_a_ (acidity constant) value [46,47].

We previously reported that PDI is *S*-nitrosylated, which decreases its enzymatic activity and leads to the accumulation of misfolded proteins, subsequently inducing ER stress [48]. *S*-nitrosylation of PDI occurs in redox-active thiols, inhibiting its enzymatic activity to result in the accumulation of polyubiquitinated proteins, an increase in ER stress, and the induction of apoptosis. Taken together, PDI is a crucial regulator for intracellular redox balance that is disrupted by excessive amounts of NO.

Intriguingly, this modification is promoted in chronic and aging-associated diseases, especially neurodegenerative diseases, during which misfolded proteins accumulate in the affected area. AD is characterized by the aggregation of amyloid-β (Aβ) plaque and neurofibrillary tangles containing abnormally phosphorylated tau [49]. These features contribute to inflammation-induced iNOS expression and then ER stress [50]. In AD, nNOS is also upregulated by the increased calcium ion (Ca^2+^) influx, which is mediated by the activation of *N*-methyl-D-aspartate (NMDA)-type glutamate receptor. The activation of iNOS and nNOS leads to the upregulation of *S*-nitrosylated (SNO)-PDI levels in AD patients [48]. Furthermore, Aβ causes neurotoxicity via oxidative stress, mitochondrial dysfunction, and aggregation of misfolded proteins [51,52]. A recent study reported that Aβ injection into the rat brain increased SNO-PDI and induced ER stress-mediated apoptosis [53]. Additionally, SNO-PDI existed in neurofibril tangles [54].

ER stress and nitrosative stress have also been implicated in the pathogenesis of PD [55]. Recently, some studies indicated that SNO-PDI contributes to oligomerization and aggregation of α-synuclein, which is a major component of Lewy body inclusion in the brain [56,57]. Also, rotenone, a pesticide, causes neurotoxicity via mitochondria complex I inhibition, and induced iNOS expression [58]. Rotenone-induced iNOS expression increases SNO-PDI formation, subsequently leading to apoptotic cell death. Moreover, we reported that the NO- and PD-inducing agent MPP^+^ caused *S*-nitrosylation of IRE1α, in addition to PDI, in human neuroblastoma cells [59]. 

Similar to AD and PD, ALS is characterized by the presence of inclusions containing aggregated proteins, such as superoxide dismutase 1 (SOD1) [60]. SNO-PDI is also increased in ALS patients and contributes to the onset and development of ALS [61]. Moreover, the SOD1 G93A mutant protein, representing a type of familial ALS gene mutation, aggregated and accumulated via SNO-PDI activity [62,63]. These results indicate that nitrosative stress induces ER stress mediated by SNO-PDI and, consequently, neuronal cell death.

### 2.3. IRE1α as a Target for NO

The amount of ER stress is monitored by transmembrane proteins, which act as ER stress sensors. The UPR is activated by three ER sensors: protein kinase R (PKR)-like endoplasmic reticulum kinase (PERK), inositol-requiring kinase 1 (IRE1), and activating transcription factor 6 (ATF6). 

PERK dimerizes or oligomerizes to induce autophosphorylation and activation. When activated, PERK phosphorylates eukaryotic initiation factor 2α (eIF2α) to induce translational repression [64]. Conversely, phosphorylated eIF2α promotes translation of activating transcription factor 4 (ATF4) to express ER chaperones, such as PDI [65]. ATF4 also activates various genes that are involved in cellular processes, such as autophagy and the antioxidant response [66]. IRE1α is activated by a similar process as PERK; it dimerizes or oligomerizes to induce autophosphorylation [2]. IRE1α reduces protein synthesis in the ER by regulated IRE1α-dependent decay (RIDD) [67]. IRE1α also catalyzes splicing of X-box binding protein 1 (XBP1) mRNA [66]. Spliced XBP1 is a transcription factor that induces the upregulation of ER chaperones and proteins involved in the ERAD pathway [68]. A third pathway, ATF6, is activated by cleavage in Golgi bodies. ATF6 translocates from the ER membrane to the Golgi body, where it is cleaved by site-1 and site-2 proteases. The cytosolic ATF6 fragment, which contains a basic-leucine zipper domain, translocates to the nucleus and induces the expression of target genes that encode ER chaperones, proteins involved in ERAD, and XBP1 [69]. 

We and others reported SNO-IRE1α formation in cysteine residues within the kinase-extension nuclease (KEN) domain that carries out a ribonuclease function [70,71]. These modifications affected XBP1 splicing but did not affect IRE1α oligomerization and phosphorylation [59,72]. In contrast to this disruption of the IRE1α–XBP1 pathway, NO does not affect the PERK and ATF6 branches [59,72]. The IRE1α–XBP1 branch acts as an anti-apoptotic pathway, whereas the PERK and ATF6 branches are implicated in cell death. Therefore, SNO-IRE1α leads to ER stress associated with neuronal cell death. These studies suggested that NO contributes to the pathology of PD mediated by *S*-nitrosylation of various target proteins. 

Type II diabetes and obesity are characterized by dysfunctional insulin signaling, which is implicated in chronic inflammation and ER stress [72,73,74]. Indeed, inflammation-associated iNOS expression is elevated in obesity and type II diabetes [75,76]. A recent study elucidated that the level of SNO-IRE1α is increased in high-fat diet induced-diabetes model mice [72]. As described above, SNO modification of IRE1α inhibits its endonuclease activity, but not phosphorylation. Interestingly, in the obese model established using liver-specific IRE1α-deficient mice, expression of IRE1α with a nitrosylation-resistant variant more effectively restored XBP1 splicing and improved glucose homeostasis than wild type IRE1α [72]. Considering that the expression of spliced XBP1 (sXBP1) also improved glucose homeostasis [72], these data suggest that the endonuclease activity of IRE1α is a critical regulator for the maintenance of glucose homeostasis. Furthermore, other studies reported that insulin resistance is acquired by inflammation, oxidative stress, and ER stress [73,75]. Also, NO can *S*-nitrosylate insulin signaling-related proteins, such as insulin receptor, insulin receptor substrate 1, and RAC-alpha serine/threonine-protein kinase (Akt), to inhibit Akt signaling [77]. These lines of evidence indicate that excessive or prolonged NO contributes not only to ER stress induction but also to insulin resistance.

## 3. HNE and ER Stress

4-Hydroxynonenal (HNE) is one of the major end products derived from the oxidation of polyunsaturated fatty acids (PUFA) by reactive oxygen species (ROS). ROS mostly affect a limited area, due to their high reactivities. Meanwhile, HNE is relatively stable, which enables it to travel a substantial distance from the site of synthesis and work as a second messenger of free radicals. HNE modifies nucleophilic residues of proteins using its three reactive groups: an aldehyde, a double bond between carbon C2 and C3, and a secondary alcohol at carbon C4. Under physiological conditions, HNE regulates several transcription factors, such as nuclear factor erythroid 2-related factor 2 (Nrf2) [78], activating protein-1 (AP-1) [79], and nuclear factor-κ B (NF-κB) [80]. HNE also modulates cell signaling pathways, including the protein kinase C [81] and epidermal growth factor receptor (EGFR) pathways [82]. However, prolonged and/or excessive oxidative stress lead to HNE accumulation, thereby disrupting the balance of regulation. High levels of HNE and HNE-modified proteins have been observed in many diseases associated with oxidative stress, such as AD [83,84], PD [85], cardiac/cerebral ischemia [86,87], and alcoholic/non-alcoholic fatty liver disease [88,89]. These data suggest that uncontrolled HNE production contributes to cellular dysfunction and disease development.

Given that loss of proteostasis was observed in these diseases [90,91,92], it has been described that HNE induces ER stress and the UPR, similar to the other electrophiles described above. Indeed, a microarray analysis of human colon cancer cells treated with HNE exhibited significant alterations of gene expression related to ER stress [93]. Nevertheless, the effects of HNE on the UPR differ among studies. HNE activated all three UPR signaling branches in human umbilical vein endothelial cells [94]. By contrast, a survey of primary rat aortic smooth muscle cells treated with HNE showed a significant increase in PERK and eIF2α phosphorylation, but robust IRE1α and ATF6 stimulation [95].

The discrepancy between the reported effects of HNE could arise from the difference between the studied species. Additionally, the capability of HNE metabolism differs in cells. Since there are several pathways for metabolizing HNE [96], cells are not all equal regarding HNE detoxification. Therefore, the response and protein modification targets of HNE-treated cells could be different in each cell line and tissue. To further understand the mechanism of HNE-induced ER stress, it is worth noting that HNE reacts with critical players of proteostasis. PDI modification by HNE inhibited its enzymatic activity, resulting in ER stress and apoptosis in endothelial cells [97]. Furthermore, heat shock protein (HSP) 70 and HSP90 were modified by HNE, which led to the inhibition of their folding functions in a rat model of alcoholic liver disease [98,99]. Interestingly, the impairment of HSP proteins is reported to promote ER stress via modulation of IRE1α activity [100,101] and downregulation of the activity of the carboxy terminus of HSP70-interacting protein (CHIP) which exerts E3 ubiquitin ligase activity associated with ERAD [102,103]. Moreover, HNE modified the 20S proteasome and suppressed its peptidase activity during coronary occlusion and reperfusion [104]. 

These studies suggest that HNE-induced disturbance of ER homeostasis could cause apoptosis and disease progression. Although further demonstration of the correlation between protein modification by HNE and disease is required, HNE might be useful as a biological marker to predict or diagnose disease given the association between high HNE concentration and pathological conditions.

## 4. MeHg and ER Stress

MeHg, a known neurotoxicant, is an environmental electrophile. Inorganic mercury is released into the air by gold mining in developing countries, or through volcanic activity. Mercury deposited in the ocean is converted into MeHg by aquatic microorganisms. MeHg has biomagnification potential and accumulates to high levels in large predatory fishes, such as whale or tuna. Humans are exposed to MeHg through ingestion of these species [105]. MeHg interacts with L-cysteine and forms the MeHg–L-cysteine complex, which conformationally mimics methionine and easily penetrates the blood–brain barrier via methionine transporters [106]. Although MeHg has been shown to cause lesions in the central nervous system, the mechanisms of MeHg-induced cell toxicity are not fully understood. In this section, we describe the molecular mechanism associated with MeHg toxicity from the viewpoint of ER stress.

The reactivity of chemical materials is evaluated according to the hard and soft acid and base (HSAB) principle. MeHg and thiolate anion are classified as a soft acid and a soft base, respectively, according to HSAB principles. MeHg strongly conjugates with the thiolate anion due to the high affinity between a soft acid and a soft base. This affinity has been shown to be greater than with various ligands of amino acids, such as a histidine residue [107]. Therefore, it has been suggested that MeHg toxicity can be attributed to covalent bonding with the thiol group of cysteine residues in proteins (*S*-mercuration) [108]. MeHg-induced disruption of microtubules and inhibition of protein synthesis have been considered as significant mechanisms involved in MeHg toxicity [109,110]. However, many recent reports mentioned this toxicity from the viewpoint of oxidative stress. For example, *S*-mercuration of manganese superoxide dismutase (MnSOD) located in mitochondria impairs MnSOD activity, resulting in ROS accumulation and mitochondrial dysfunction [111]. Furthermore, glutathione (GSH), which is known to contribute to the elimination of MeHg by GSH conjugation, decreases after high exposure to MeHg, subsequently inducing oxidative stress [112]. 

UPR, especially the IRE1α branch, may be linked to the cytotoxicity of MeHg-derived oxidative stress. Apoptosis signal-regulating kinase 1 (ASK1) is a part of the mitogen-activated protein kinase pathway, which is activated in response to various stresses and inactivated by forming a complex with thioredoxin (Trx) at steady-state. However, oxidized Trx dissociates from ASK1 under oxidative stress, resulting in apoptosis induced by activated ASK1 [113]. In a previous study, MeHg-derived accumulation of ROS was shown to evoke ASK1 activation and cause cell death [114]. Moreover, ASK1 has been shown to interact with the IRE1α–TRAF2 complex and induce apoptosis under ER stress [115]. These findings suggest that MeHg-induced cell death is derived from ER stress following induction of oxidative stress [114]. This theory is supported by another research group, which found that Trolox, a known ROS scavenger, inhibits MeHg-induced ER stress [116]. Conversely, we have shown that MeHg induced oxidative modification of PDI, similar to NO [117]. MeHg attenuates the enzymatic activity of PDI by *S*-mercuration and results in ER stress [117]. Taken together, MeHg elicits ER stress from diverse approaches.

Caspase-12 belongs to the caspase family and is activated by ER stress [118]. MeHg is thought to trigger apoptosis via induction of ER stress caused by caspase activation [114,116]. Additionally, we found that *S*-mercuration of IRE1α interferes IRE1α–XBP1 signaling (Figure 1 [119]). Activation of IRE1α–TRAF2–ASK1 pathway was also observed in XBP1-deficient cells [120]. These findings indicate that inhibition of the IRE1α–XBP1 pathway strongly accelerates cell death. 

MeHg impairs the central nervous system, suggesting that MeHg is relevant to the onset of neurodegenerative diseases, such as AD, PD, and ALS [121,122,123,124]. Additionally, it has been reported that MeHg affects fetal neurodevelopment via prenatal exposure to MeHg [125]. These nervous system diseases are highly related to ER stress [126,127,128,129]. Hence, MeHg-induced ER stress may contribute to the onset of these nervous system diseases. The effect of MeHg on human health is a relevant issue because humans continuously uptake MeHg via fish consumption. Therefore, elucidating the molecular mechanism of MeHg toxicity remains important.

## 5. Conclusions

In addition to the RES mentioned above, other RES, such as 1,4-naphthoquinone [130], acrolein [131,132,133], and electrophilic prostaglandins [134,135], have also been associated with ER stress. These lines of evidence support the idea that RES are the key factor controlling ER stress responses. Although further studies are required to fully elucidate each mechanism, targeting or inversely making use of RES modulation of ER stress might be the next generation of therapeutic methodology for associated diseases.

## Abbreviations

AD	Alzheimer’s disease
Akt	RAC-alpha serine/threonine-protein kinase
ALS	Amyotrophic lateral sclerosis
AP-1	Activating protein-1
ASK1	Apoptosis signal-regulating kinase 1
ATF6	Activating transcription factor 6
Aβ	Amyloid-β
Ca^2+^	Calcium ion
CHIP	Carboxy terminus of HSP70-interacting protein
EGFR	Epidermal growth factor receptor
eIF2α	Eukaryotic translation factor 2α
eNOS	Endothelial NOS
ER	Endoplasmic reticulum
ERAD	ER-associated degradation
GSH	Glutathione
HNE	4-Hydroxynonenal
HSAB	Hard and soft acid and base
HSP70	Heat shock protein 70
HSP90	Heat shock protein 90
iNOS	Inducible NOS
IRE1α	Inositol-requiring kinase 1α
JNK	c-Jun N-terminal kinase
KEN	Kinase-extension nuclease
MeHg	Methylmercury
MnSOD	Manganese superoxide dismutase
MPP+	1-Methyl-4-phenylpyridinium iodide
NF-κB	Nuclear factor-κ B
NMDA	*N*-Methyl-D-aspartate
nNOS	Neuronal NOS
NO	Nitric oxide
NOS	Nitric oxide synthase
Nrf2	Nuclear factor erythroid 2-related factor 2
PUFA	Polyunsaturated fatty acids
PD	Parkinson’s disease
PDI	Protein disulfide isomerase
PERK	PKR-like endoplasmic reticulum kinase
PKR	Protein kinase R
RES	Reactive electrophile species
RIDD	Regulated IRE1α-dependent decay
ROS	Reactive oxygen species
SNO	*S*-Nitrosylated
SOD1	Superoxide dismutase 1
TRAF2	TNF receptor-associated factor 2
Trolox	6-Hydroxy-2,5,7,8-tetramethylchroman-2-carboxylic acid
Trx	Thioredoxin
UPR	Unfolded protein response
XBP1	X-box binding protein 1
